# Promoter prediction and annotation of microbial genomes based on DNA sequence and structural responses to superhelical stress

**DOI:** 10.1186/1471-2105-7-248

**Published:** 2006-05-05

**Authors:** Huiquan Wang, Craig J Benham

**Affiliations:** 1 UC Davis Genome Center, University of California, One Shields Avenue, Davis, CA 95616, USA

## Abstract

**Background:**

In our previous studies, we found that the sites in prokaryotic genomes which are most susceptible to duplex destabilization under the negative superhelical stresses that occur *in vivo *are statistically highly significantly associated with intergenic regions that are known or inferred to contain promoters. In this report we investigate how this structural property, either alone or together with other structural and sequence attributes, may be used to search prokaryotic genomes for promoters.

**Results:**

We show that the propensity for stress-induced DNA duplex destabilization (SIDD) is closely associated with specific promoter regions. The extent of destabilization in promoter-containing regions is found to be bimodally distributed. When compared with DNA curvature, deformability, thermostability or sequence motif scores within the -10 region, SIDD is found to be the most informative DNA property regarding promoter locations in the *E. coli *K12 genome. SIDD properties alone perform better at detecting promoter regions than other programs trained on this genome. Because this approach has a very low false positive rate, it can be used to predict with high confidence the subset of promoters that are strongly destabilized. When SIDD properties are combined with -10 motif scores in a linear classification function, they predict promoter regions with better than 80% accuracy. When these methods were tested with promoter and non-promoter sequences from *Bacillus subtilis*, they achieved similar or higher accuracies. We also present a strictly SIDD-based predictor for annotating promoter sequences in complete microbial genomes.

**Conclusion:**

In this report we show that the propensity to undergo stress-induced duplex destabilization (SIDD) is a distinctive structural attribute of many prokaryotic promoter sequences. We have developed methods to identify promoter sequences in prokaryotic genomes that use SIDD either as a sole predictor or in combination with other DNA structural and sequence properties. Although these methods cannot predict all the promoter-containing regions in a genome, they do find large sets of potential regions that have high probabilities of being true positives. This approach could be especially valuable for annotating those genomes about which there is limited experimental data.

## Background

As the number of completely sequenced microbial genomes grows, the need for efficient annotation tools becomes more acute. Gene-finding programs such as GeneMark or Glimmer [1,2] can predict protein coding regions at a generally high level of accuracy. However, there also are genes encoding rRNA, tRNA and small non-coding RNAs in prokaryotic genomes, which these methods may not always find. The precise locations of translation and transcription start sites also need to be identified. Better understanding of the attributes associated with promoters, in addition to shedding light on the basic mechanisms by which they function, will also assist in identifying these sites within genomic sequences.

Promoter prediction in prokaryotic genomes presents unique challenges owing to their organizational properties. First, gene densities are very high in prokaryotes – 89% of the base pairs in the *E. coli *genome are in open reading frames (ORFs). Neighboring genes may have very short intergenic regions; in some cases their coding regions even overlap. Further, the operon structure, in which multiple genes are transcribed as a single transcription unit, means that not all genes require their own promoters. In order for bacteria to thrive in different environments, their genomic sequences are highly adaptive within genomes and highly diversified across genomes. This makes it difficult to detect conserved regulatory sites within and across genomes by sequence homology. These factors have complicated the search for the determinants of promoter activity in prokaryotes.

Prokaryotic promoters are known to contain conserved sequence motifs, which may be represented either as consensus sequences or by position-specific score matrices (PSSMs) [3]. For example, most *E. coli *K12 promoters contain approximately conserved sequence elements in their -35 and -10 regions [4-6]. The -10 motif is essential for transcription initiation, while the -35 motif is dispensable for some promoters. Other sequence features of *E. coli *K12 promoters include the A+T rich UP element located around position -50 [7]. Most of the promoter prediction programs thus far developed search sequences for conserved -10 motifs, and in some cases also include -35 motifs [8,9]. These methods commonly suffer from high false positive rates.

Local separation of the DNA duplex within promoter regions is a critical step in transcriptional initiation for both prokaryotes and eukaryotes. This step must be highly regulated, but does not occur as a strictly thermal melting. Instead, the untwisting torsional stresses imposed on genomic DNA by *in vivo *negative superhelicity destabilize the DNA duplex in specific regions, and thereby facilitate local strand opening. We have developed statistical mechanical methods to analyze this stress-induced DNA duplex destabilization (SIDD) within complete chromosomes. For a specified level of superhelicity, we calculate two quantities for each base pair – the probability of its opening, and the incremental free energy *G*(*x*) needed to force it to be always open under these conditions [10,11]. A small number of specific sites in genomic DNA are predicted by SIDD analysis to have a high propensity to melt under normal physiological conditions. We have demonstrated that these SIDD sites in the *E. coli *K12 genome are statistically significantly associated with intergenic regions that are known or inferred to contain promoters. It is found that many – but not every – documented promoter contains a strong SIDD site. Further, SIDD sites also occur at frequencies much below expectation in coding regions [12]. This pattern of SIDD site distribution has recently been confirmed to occur in many other prokaryotic genomes [13]. This suggests that SIDD properties may be used to identify and investigate promoter-containing regions in prokaryotic genomes.

In this report we show that SIDD is a distinct structural property of promoter regions that cannot be identified by sequence conservation. There is no one-to-one correspondence between local attributes of the sequence and the extent of destabilization, and no unique sequence motifs are involved. When compared with other known DNA structural properties and with -10 motif scores, SIDD properties are found to be the best discriminator for distinguishing promoters from non-promoters. When SIDD was either the sole predictor or was combined with other features in a promoter prediction program, high levels of sensitivity and specificity were achieved. We present two approaches for annotating promoter locations in sequenced prokaryotic genomes.

## Results and discussion

### SIDD is a distinct structural property in promoter regions that cannot be captured by sequence conservation

We first performed a SIDD analysis of the entire *E. coli *K12 genome, as described in the Methods section. Then three sets of regions were selected according to their transcriptional properties, and their SIDD attributes were compared. To construct the first set we randomly selected 500 documented transcription start sites (TSS) from the 927 that are annotated in the Regulon database for the *E. coli K12 *genome [14]. For each of these TSSs, we found the 1001 bp sequence centered on that TSS. The regions immediately upstream from these TSSs were considered to be promoters, which hence would be contained in these regions. For comparison we selected two other sets for analysis, each also containing 500 sequences that are 1001 bp long, that are chosen to contain few or no promoters. Each sequence in the second set starts at one of the TSSs chosen above, and extends 1001 bp in the direction of transcription. This is called the coding set of sequences. The third set consists of 500 randomly selected sequences, each 1001 bp long, centered on an intergenic region separating convergently oriented genes. This set, which we call CON, may contain parts of ORFs, but is inferred not to contain promoters. We also constructed a fourth set of random sequence DNA as follows. We randomly shuffled the entire *E. coli *K12 genome to preserve the mononucleotide compositions, and performed a SIDD analysis on the entire resulting sequence. Then a set of 500 sequences, each 1001 bp long, was chosen at random from this shuffled genome, and their SIDD attributes were compared with those of the sequences in the other three sets.

**Figure 1:**
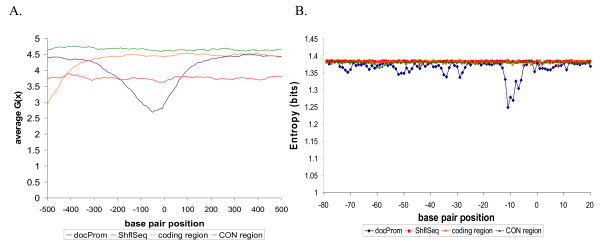
A. The average value of *G*(*x*) at each position in each of the four sets described in the text. Promoter regions in *E. coli *K12 are generally more destabilized than are either coding or CON regions. The most destabilized site is at position -49 relative to the transcription start site (TSS). B. The Shannon entropy is calculated in promoter-containing regions (blue) and in non- promoter-containing regions (red) for *E. coli *K12. The -10 regions show a slight increase of sequence conservation relative to other locations.

First, for each of these sets we determined the average value of the destabilization free energy *G*(*x*) that occurs at each position within the 1001 bp sequence. (The destabilization free energy *G*(*x*) corresponds to the incremental free energy needed for the base pair at position *x *to always remain open. Highly destabilized sites have low values of *G*(*x*); sites that are open in all low energy states can even have negative values.) These calculated position-specific average values are shown in Figure 1A. On average, the sequences containing promoters are more destabilized than either the coding sequences or the CON sequences that may not contain promoters. These results agree with those from our previous analysis, which showed strong SIDD sites to be statistically significantly associated with intergenic regions whose bounding ORFs are either divergently or tandemly oriented, and hence may contain promoters or other transcriptional regulatory elements [12]. The lowest average value of *G*(*x*) occurs around position -49 relative to the TSS, a region previously identified as the UP element in some *E. coli *K12 promoters. Statistical analysis shows the regions between positions -174 and +57 are significantly destabilized (p < 0.001) when compared with either of the two sets that do not contain promoters, or with the randomly shuffled sequences.

In what follows we designate the 100 bp sequence from positions -80 to +20 relative to each of the 500 chosen TSSs as containing a promoter. (This is 100 base pairs, there being no base pair that is given the position 0.) These 500 promoter sequences were aligned at their TSSs, and their sequence conservation was measured by Shannon's entropy [15]. The result is shown in Figure 1B. As expected, a slight entropy decrease (corresponding to slightly increased sequence conservation) was observed between positions -7 and -12, which are their -10 regions. However, there was no significant entropy decrease at the -35 regions, indicating that on average the local sequence is not more conserved at this location than elsewhere in these promoters. It is known that the -35 motif is dispensable for so-called "extended -10" sigma 70 promoters. Strikingly, the region around -49 bp where the maximal average destabilization was found also shows no increase in sequence conservation. This indicates that SIDD properties and -10 motifs are fundamentally different attributes of promoters; one is tied directly to the base sequence but the other is not.

A recent paper that presented a promoter prediction method based on thermostability in promoter regions reported that the region from -20 to -6 was much less stable than were other, non-promoter locations [16]. It is possible that the decreased thermostability in this region was partly due to the conserved -10 sequence motif, which is A+T-rich.

**Figure 2:**
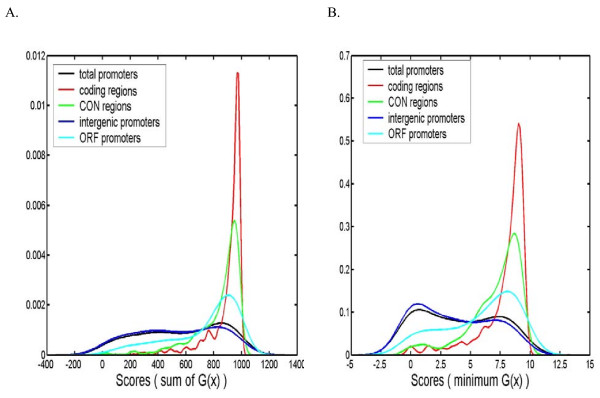
The probability distributions of destabilization (SIDD) properties are shown for 100 bp long promoter, coding, and CON regions. We also partition the promoters from all known TSSs into those that are intergenic and those that overlap coding regions. (A) The SIDD parameter here is the sum over all 100 positions of the destabilization energy *G*(*x*); (B) The parameter used here is the minimum value *G*_*m *_of *G*(*x*). Because the numbers of intergenic and coding promoters are different, these curves are plotted as probability density functions. In consequence, the area under each curve sums to 1, a choice which facilitates comparisons between different sized sets.

This result emphasizes the fact that SIDD properties are fundamentally different from thermostability. SIDD does not depend only on the local thermal properties of the DNA sequence. The energies that govern SIDD are the differences between the energy cost of strand separation for the specific base pairs involved, and the energy benefit from the fractional relaxation of the superhelical stress this transition provides. The thermal energy only relates to the cost half of this relationship. Moreover, superhelical stresses couple together all the base pairs that experience them, so whether SIDD melting occurs at any specific location depends on how well that site competes with all others that feel this stress. This means a site can open at one level of stress, then re-close coupled to opening elsewhere as the stresses are increased. (See Fig 2 of the reference [11], where both sites have the same thermodynamic stability.) This type of complicated, nonlinear behavior does not occur in thermal melting, and cannot be predicted only from the thermal properties of the sequence.

### Construction of training and test sets

All the analyses that follow used three training sets, each of which contains 500 sequences that are 100 bp long. The promoter-containing training set, described above, consists of sequences spanning positions -80 to +20 relative to each of the 500 selected TSSs. In each of the 500 CON sequences described above we selected the central 100 bp, which are centered in the middle of their intergenic regions. As these regions are short and separate convergently transcribing ORFs, they are inferred not to contain promoters. Henceforth we describe this as the CON training set. The third training set consists of the 100 base pair sequences between position +300 and +399 within each of the 1001 bp coding sequences described above. This set, which is sometimes referred to as the coding training set, also is inferred not to contain promoters.

We also construct three test sets, each having the same transcriptional properties as its corresponding training set. These test sets also contains sequences of length 100 bp, constructed in the same way as were the training sets. The promoter-containing test set was constructed from the 427 experimentally characterized TSSs that were not used to construct the training set. The coding test set consists of the sequences between position +300 and +399 relative to each of these 427 TSSs. The CON test set consists of 427 different 100 bp sequences, each centered in the middle of an intergenic region that separates convergently transcribing ORFs. These regions were randomly chosen from among those that were not used to make the CON training set.

Thus we have three types of training sets of equal size, and three types of test sets, also of equal size (but a bit smaller than the training sets). Each of these three set types has distinct transcriptional attributes. The first contains promoters, the second contains coding sequences but not promoters, and the third contains terminal intergenic sequences that also do not contain promoters. Some of the analyses whose results are described below do not involve test sets. In those cases the larger, 500 sequence training sets were analyzed. However, in procedures where parameters had to be developed, the training sets were used for that purpose. Then the procedure with those parameter values was applied to the test sets.

We know the SIDD properties of each sequence in these sets, as these were determined through the whole genome SIDD analysis described in the Methods section. Next, we compare the values of a variety of attributes in promoter-containing regions to their values in each of the two types of non-promoter-containing regions. We describe each in turn.

### SIDD energy levels are bimodally distributed among promoter sequences

We first examined two SIDD properties of the sequences in each of these types of regions. These are the sum Σ of the *G*(*x*) values for the 100 base pairs in each sequence in the set, and the minimum value *G*_*m *_attained by *G*(*x*) in that sequence. The probability distributions of these quantities over each of the three sets are shown in Figure 2. (There being no training involved in this procedure, the training sets were used in these analyses.) The majority of the non-promoter sequences have high values of both Σ and *G*_*m*_, indicating that they remain stable under superhelical stress. However, the distributions of both parameters within promoters are bimodal. It appears that two sub-populations of promoter sequences can be distinguished according to their SIDD properties, one group being highly destabilized and the other less so.

Because we previously showed that strong SIDD sites are closely associated with promoter-containing intergenic regions and avoid coding regions [12], we wanted to test whether this bimodal character could be partially attributed to promoter location. For this purpose we separated the full set of 927 experimentally characterized TSSs into those that are intergenic and those that occur within ORFs. For each we chose the 100 base pair segments from positions -80 to +20 as their promoter-containing regions. We found the Σ and *G*_*m *_values for each of these regions, considering the intraORF-TSS and intergenic-TSS sets separately. More than 80% of these 927 TSSs are located in intergenic regions, so these sets have substantially different sizes. For this reason we plot in Figure 2 the probability distributions of each score for each of these two additional sets. As shown in the figure, there is some enrichment of promoters with intraORF TSSs in the non-destabilized population. But because the number of these promoters is small, the bimodal character of the intergenic-TSS promoter set is not much different from that of the 500 sequence promoter-containing training set that contains both types.

The observed bimodal distribution of SIDD properties in promoters may reflect the complexity of transcriptional regulation, suggesting that superhelical destabilization may be needed to initiate transcription from some promoters, but not others. SIDD in highly destabilized promoters may be directly involved in the mechanism of open complex formation. However, one cannot rule out the possibility that SIDD also could be involved in regulating more stable promoters. In the present analysis we confined our attention to the 100 bp sequence from -80 bp to +20 bp. But SIDD sites further upstream are known to play central roles in specific mechanisms of transcriptional regulation. An example is the IHF-mediated transcriptional activation of the promoter governing the expression of the *E. coli *ilvGMEDA operon [17]. In the absence of IHF binding, negative superhelicity opens the SIDD region, which is located upstream from position -90. IHF binding forces this region back to B-form, so the superhelical stresses open the next most easily destabilized site, which is in the -10 region. This regulatory mechanism involves a binding-induced transmission of destabilization from the binding site into the promoter. SIDD clearly plays a central role in this mechanism, even though the regulatory SIDD site is not at the promoter. A similar mechanism was also observed to regulate the LeuV operon, which involved the binding of *fis *to a SIDD site [18].

### SIDD is more capable than other structural or sequence properties of distinguishing promoters from non-promoter sequences in *E. coli *K12

Other research groups have reported that several other types of structural parameters also appear to be distributed differently between promoters and non-promoters. These include DNA intrinsic curvature, protein-induced deformability, and thermodynamic stability [19-21]. To compare these attributes with SIDD properties, we used published methods to calculate each property for the entire genome as described in the Methods section, then examined their values in the test and training sets.

**Figure 3:**
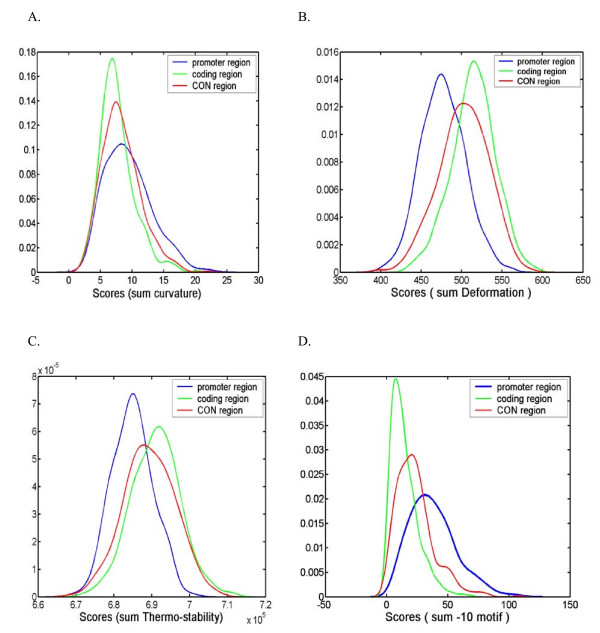
Probability distributions of the sums of the scores for (A) curvature, (B) deformability, (C) thermostability, and (D) the sum of all -10 motif scores found in promoter regions, coding regions and CON regions. These are plotted the same way as was used in Figure 2.

We determined the sums, and the maximum and minimum values, of each of these quantities over each of the 100 bp training sets described above. The distributions of the sums of these parameters in promoters, and in the coding and CON non-promoter training sets, are shown in Figure 3ABC. Overall, promoter regions tend to be slightly more curved, less flexible for protein binding, and less stable under thermal fluctuations than are non-promoter regions. However, these feature differences are much less dramatic than those shown in Figure 2 for SIDD properties. The corresponding graphs for the maxima and minima of these parameters are less informative, but show similar trends. (Data not shown.)

Position-specific score matrices (PSSMs) are frequently used to represent conserved motifs, such as protein binding sites. PSSM methods based either on the -10 motif or on motifs from both the -10 and -35 regions have been used to find putative sigma factor binding sites, presumably as signals for promoters [8,9]. By aligning the promoter sequences in the training set at their TSSs, we derived a log-odds PSSM for the -10 motif (from -6 to -13) in the promoters, using the method described in [3]. When this PSSM was used to search our promoter and non-promoter test sequences, we found that promoters contain higher densities of high-scoring PSSM motifs than do either the coding or the CON classes of non-promoters (figure 3D, and other data not shown). However, there are substantial numbers of these motifs in all three sets. But the average *density *of high-scoring PSSM motifs is approximately twice as high in promoter-containing sequences as in those that do not contain promoters. So the sum of the motif scores over a window may be a useful discriminator of promoters. But the exact locations of -10 motifs alone may not be useful for inferring either transcription start sites or promoter locations.

We have examined the distributions of each of these five properties (SIDD, curvature, deformability, thermodynamic stability, and -10 motif scores) for each set of promoter and non-promoter sequences in each of two cases – first using the summed variable and then (except for -10 scores) its extreme value in the region. (As promoters are thought to have high intrinsic curvature we used maxima of this parameter. For SIDD properties, deformation energy and thermodynamic stability we used minima.) In each case the statistical significances of the differences found between the promoter-containing training set and each of the two non-promoter containing sets were calculated using the Kolmogorov-Smirnov test [22]. The results of these statistical analyses are shown in Table 1. For all parameters the distributions of the summed variables show statistically significant differences between promoters and either of the non-promoter sets. Among these, the SIDD property shows the highest significance level. The distribution of extreme values remains highly significant for SIDD, but is much less so for the other parameters. In fact, only the difference of the maximum values of curvature between promoter and non-promoter sets retains significance at the 95% level, while the differences of minimum values of either deformation energy or thermostability between promoters and non-promoters are not significant. Thus, among these variables, SIDD is the most informative for differentiating promoters from non-promoters, followed by -10 motif scores.

### SIDD alone outperforms other parameters in detecting promoter sequences in *E. coli K12 *and in *Bacillus subtilis*

**Figure 4:**
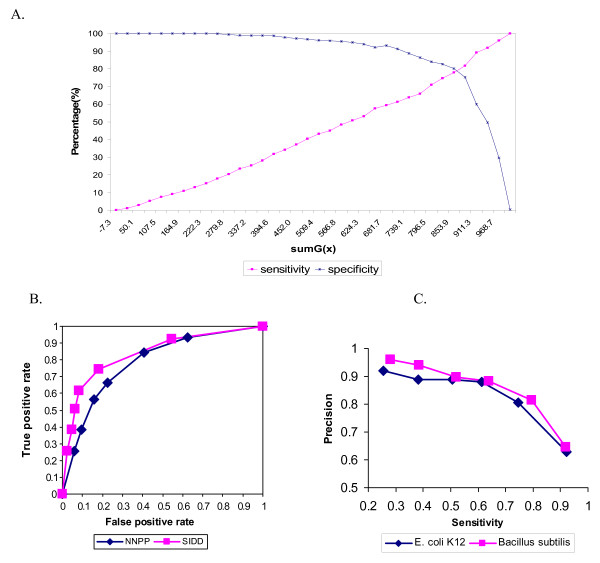
(A) The sensitivity (true positive rate) and specificity (1 – false positive rate) achieved in classifying 1000 *E. coli *K12 sequences into promoters and non-promoters, based on their sum of *G*(*x*) parameters. The graph shows how the sensitivity and specificity depend on the threshold for regarding a sequence as a promoter. Of the 1000 sequences analyzed here, half are promoters and half are not. (B) ROC curves comparing the performance of SIDD vs NNPP at identifying promoters in *E. coli *K12. (C) The performance of our SIDD-based promoter discriminator is comparable for sequences in *E. coli *K12 and in *Bacillus subtilis*.

In order to determine the values of the SIDD parameters Σ and *G*_*m *_that most reliably discriminate promoter from non-promoter sequences, we first chose a series of values spanning the ranges of their distributions. Each value was regarded as a threshold, so in this analysis a sequence would be classified as containing a promoter or not, solely based on whether or not its sum Σ or minimum value *G*_*m *_fell below this threshold. In this way we placed all the 100 bp sequences in the three training sequences in the *E. coli *genome into promoter or non-promoter bins, then calculated the true and false positive rates achieved when that threshold was used. This was done for each value of Σ or *G*_*m *_in the series, and also for each pair (Σ, *G*_*m*_) of values. Figure 4A shows the sensitivities and specificities as the threshold of Σ is varied. (Sensitivity = true positive rate; specificity = 1- false positive rate.) Similar results are found for *G*_*m *_and also for pairs (Σ, *G*_*m*_) (Representative data presented in Supplementary Information [see Additional file 1].) As this figure shows, the sensitivity increases monotonically with threshold, while the specificity decreases monotonically. However, the specificity is essentially unity until a substantial threshold, around Σ = 280 in this case. In this way a range of threshold values of the parameters Σ and *G*_*m *_were obtained that provide high true positive rates and low false positive rates.

We note that sensitivities of 25% to 30% can be achieved with very small false positive rates, but that higher sensitivities have an increasing cost in terms of decreased specificity. This corresponds to our previous observation that, although strongly destabilized sites are highly concentrated at promoters, not all promoters are destabilized. This fact also is reflected in the bimodal distributions of SIDD parameters at promoters that were documented in Figure 2. However, at this modest level of sensitivity the specificity is so high that false positives are very few. This will be shown to be an important attribute for promoter prediction, as most other methods suffer from high false positive rates. Moreover, for purposes of comparing methods the important attribute is the true positive rate *versus *the false positive rate, as described below.

We compared the performances on the test sets of these optimized SIDD predictors with that of the publicly available NNPP promoter prediction program. Although NNPP was originally developed to predict core promoter regions in the *Drosophila melanogaster *genome [23], it was also trained on a set of documented promoter sequences in *E. coli *K12. (We note that the number of *E. coli *K12 promoter sequences used to train NNPP was about half the size of the training set used in our study.) NNPP is a neural network-based computer program that uses a time-delay architecture to incorporate structural and compositional properties of promoter sequences. By setting its stringency between 0.1 and 0.9, we obtained a range of NNPP predictions regarding whether each sequence in each of our sets either did or did not contain a promoter. The true positive and false positive rates achieved were calculated for each level of stringency. Since our SIDD method detects promoter-containing regions without pinpointing the TSS while NNPP predicts TSSs, care must be taken to calculate these rates in equivalent manners. If NNPP predicts that a sequence contains a TSS, no matter where it is, for the purposes of comparing with the SIDD results that sequence was considered to contain a promoter.

The performance characteristics of the SIDD predictor and NNPP were compared using a ROC (receiver operating characteristic) curve, which graphs the true positive rate *vs *the false positive rate achieved by each method for several values of SIDD parameter thresholds and NNPP stringencies. The results are shown in Figure 4B. The better the predictor the more the curve moves towards the vertical axis, having higher true positive rates and lower false positive rates. At a given false positive rate, SIDD always predicted more true positives than did NNPP. For example, SIDD correctly predicted 74.6% of the real promoters with a false positive rate of 18%. When NNPP correctly predicted 66.4% of the real promoters, it had at 22.4% false positive rate. The area under the ROC is a convenient way of comparing classifiers. If the area under the ROC curve is unity, the classifier is perfect – it gives a 100% true positive rate with no false positives. Conversely, a method with no classifying power will have a diagonal curve with area 0.5 – it cannot distinguish true from false positives, giving both at the same rates. The SIDD predictor curve in Figure 4B has area 0.831, while the NNPP curve has area 0.785. By this criterion SIDD has better predictive power than does NNPP.

The pattern of SIDD distribution found in *E. coli *K12, in which strongly destabilized sites are concentrated at promoters and avoid coding sequences, also occurs in other prokaryotic genomes [24]. So we also applied our methods to *Bacillus subtilis*, the only prokaryote from a different phylum than *E. coli *that has extensive experimental annotation of promoters. We developed training and test sets as described above for *E. coli*, and examined their SIDD properties as above. (The SIDD properties again were calculated in a whole genome analysis.) In this organism the most extreme level of average destabilization also occurs at position -49 relative to the TSSs. The sequences at this site are not as conserved as that of -10 regions, as was shown in Figure 1A to occur in *E. coli *K12. A bimodal distribution of SIDD properties also is found in *Bacillus subtilis *promoter regions, just as it is in *E. coli K12*. (These results on *Bacillus subtilis *are presented in the Suppplementary Information [see Additional file ]1.) When SIDD alone was used to differentiate promoter-containing from non-promoter-containing sequences in *B. subtilis*, it demonstrated comparable performance to that achieved for *E. coli *K12, as is shown in Figure 4C. Despite of the different nucleotide compositions of the genomes of *E. coli *K12 and *Bacillus subtilis *(the AT/GC ratio is 0.968 in *E. coli *K12, and 1.298 in *B. subtilis*), and the large evolutionary distance separating them, SIDD consistently predicted promoter and non-promoter sequences in both organisms with comparably high precisions and low false positive rates. (Precision is the fraction of all positive predictions that are true.) Thus, SIDD characteristics may be capable of detecting promoter-containing sequences in many prokaryotic genomes.

A recent paper that used thermostability as a promoter predictor also claimed that their method was likely to be applicable to different microorganisms [16]. Both our method and theirs have been tested using the same sources of experimental TSSs from *E. coli *K12 and *Bacillus subtilis*. Since their program was not publicly available, we tried to compare the performances of both methods by plotting our figure 4C in a way equivalent to that of their Figure 9, which showed the precision of their method. The definitions of precision and sensitivity used in our figure 4C are the same as those defined there. (See the Methods section for precise definitions.) As the sensitivity of the thermostability method increased from 20% to 90%, its published precision decreased dramatically – from about 72% to about 37% for *E. coli *K12, and from about 82% to 27% for *B. subtilis*. In contrast, the predicted precisions achieved by our SIDD-based method at all levels of sensitivity remained higher than 62% for both *E. coli *K12 and *B. subtilis*. Even at sensitivities below 30%, precisions exceeding 90% were achieved for both organisms. These high precisions are achieved because the SIDD method has very low false positive rates. So while it may find only a fraction of the actual promoters, those it does identify have high probabilities of being true. As can be seen by comparing Figure 4C here with Figure 9 in the paper describing the thermostability method [16], the SIDD technique significantly outperformed that technique. These results further support the claim that SIDD is a better discriminator of promoter sequences than is thermostability.

### SIDD can predict with high confidence the set of promoters that have strong stress-induced destabilization properties

A substantial number of experimentally characterized promoters are required to construct training sequences for most sequence-based promoter prediction programs. Sufficiently large documented promoter sets are only available for the *E. coli *K12 and *B. subtilis *bacterial genomes. One way to circumvent this shortage is to *ab initio *predict promoters by also identifying the distinct sequence and/or structural attributes with which they are associated. In our previous and current studies we have shown that strongly destabilized SIDD regions in the *E. coli *K12 genome are statistically highly significantly associated with promoters [12]. Since the SIDD profile of a genome is directly calculated from its primary sequence, SIDD properties can be used to predict promoters, even in cases where training sequences are limited. The following is a demonstration of how SIDD properties can be used to estimate the probability that a 100 bp fragment in the *E. coli *K12 genome contains a promoter. For this purpose we use the sum SIDD parameter Σ, although similar results may be obtained with the minimum value *G*_*m*_. This approach can be applied to any completely sequenced bacterial genome, once its SIDD profile has been calculated.

Figure 4A shows, for any value of the threshold (*T*) of Σ, the true positive rate for a 100 bp promoter sequence (P) to have the sum *D *of its SIDD energies satisfy *D *<*T*. This gives the probability *p*(*D *<*T *|P) that this sum satisfies the threshold inequality, given that the sequence involved contains a promoter. From the false positive rate, also found from Fig 4A, one can determine the probability *p*(*D *<*T *|~P) that a sequence which is not a promoter (~P) satisfies the inequality *D *<*T*. In order to use SIDD for promoter prediction, one must estimate the *a posteriori *probabilities that a 100 bp region in the genome either is or is not a promoter, given that the sum *D *of its SIDD energies satisfies *D *<*T*. These are *p*(P| *D *<*T*) and *p*(~P | *D *<*T*), respectively. According to Bayes' theorem, these quantities can be obtained by

p(P|D<T)=p(D<T|P)p(P)p(D<T);     (1a)
 MathType@MTEF@5@5@+=feaafiart1ev1aaatCvAUfKttLearuWrP9MDH5MBPbIqV92AaeXatLxBI9gBaebbnrfifHhDYfgasaacH8akY=wiFfYdH8Gipec8Eeeu0xXdbba9frFj0=OqFfea0dXdd9vqai=hGuQ8kuc9pgc9s8qqaq=dirpe0xb9q8qiLsFr0=vr0=vr0dc8meaabaqaciaacaGaaeqabaqabeGadaaakeaacqWGWbaCcqGGOaakcqWGqbaucqGG8baFcqWGebarcqGH8aapcqWGubavcqGGPaqkcqGH9aqpdaWcaaqaaiabdchaWjabcIcaOiabdseaejabgYda8iabdsfaujabcYha8jabdcfaqjabcMcaPiabdchaWjabcIcaOiabdcfaqjabcMcaPaqaaiabdchaWjabcIcaOiabdseaejabgYda8iabdsfaujabcMcaPaaacqGG7aWocaWLjaGaaCzcamaabmaabaGaeGymaeJaemyyaegacaGLOaGaayzkaaaaaa@5081@

and p(~P|D<T)=p(D<T|~P)p(~P)p(D<T);     (1b)
 MathType@MTEF@5@5@+=feaafiart1ev1aaatCvAUfKttLearuWrP9MDH5MBPbIqV92AaeXatLxBI9gBaebbnrfifHhDYfgasaacH8akY=wiFfYdH8Gipec8Eeeu0xXdbba9frFj0=OqFfea0dXdd9vqai=hGuQ8kuc9pgc9s8qqaq=dirpe0xb9q8qiLsFr0=vr0=vr0dc8meaabaqaciaacaGaaeqabaqabeGadaaakeaacqWGWbaCcqGGOaakcqGG+bGFcqWGqbaucqGG8baFcqWGebarcqGH8aapcqWGubavcqGGPaqkcqGH9aqpdaWcaaqaaiabdchaWjabcIcaOiabdseaejabgYda8iabdsfaujabcYha8jabc6ha+jabdcfaqjabcMcaPiabdchaWjabcIcaOiabc6ha+jabdcfaqjabcMcaPaqaaiabdchaWjabcIcaOiabdseaejabgYda8iabdsfaujabcMcaPaaacqGG7aWocaWLjaGaaCzcamaabmaabaGaeGymaeJaemOyaigacaGLOaGaayzkaaaaaa@550F@

respectively.

For the purposes of this calculation we consider 100 bp segments in the *E. coli *K12 genome, and seek the probabilities that they contain promoters, given their SIDD properties. The number *n*_*d *_of DNA segments having *D *<*T *can be calculated directly from the SIDD profile of the genome. The number *n*_*p *_of segments that contain promoters is not known, so illustrative calculations can be made using various values for this quantity. Then the probabilities *p*(*D *<*T*) and *p*(P) can be estimated as the fractions of base pairs in the genome that lie in these two types of regions. These are

p(D<T)=100ndN,     (2a)
 MathType@MTEF@5@5@+=feaafiart1ev1aaatCvAUfKttLearuWrP9MDH5MBPbIqV92AaeXatLxBI9gBaebbnrfifHhDYfgasaacH8akY=wiFfYdH8Gipec8Eeeu0xXdbba9frFj0=OqFfea0dXdd9vqai=hGuQ8kuc9pgc9s8qqaq=dirpe0xb9q8qiLsFr0=vr0=vr0dc8meaabaqaciaacaGaaeqabaqabeGadaaakeaacqWGWbaCcqGGOaakcqWGebarcqGH8aapcqWGubavcqGGPaqkcqGH9aqpdaWcaaqaaiabigdaXiabicdaWiabicdaWiabd6gaUnaaBaaaleaacqWGKbazaeqaaaGcbaGaemOta4eaaiabcYcaSiaaxMaacaWLjaWaaeWaaeaacqaIYaGmcqWGHbqyaiaawIcacaGLPaaaaaa@40EA@

and p(P)=100npN,     (2b)
 MathType@MTEF@5@5@+=feaafiart1ev1aaatCvAUfKttLearuWrP9MDH5MBPbIqV92AaeXatLxBI9gBaebbnrfifHhDYfgasaacH8akY=wiFfYdH8Gipec8Eeeu0xXdbba9frFj0=OqFfea0dXdd9vqai=hGuQ8kuc9pgc9s8qqaq=dirpe0xb9q8qiLsFr0=vr0=vr0dc8meaabaqaciaacaGaaeqabaqabeGadaaakeaacqWGWbaCcqGGOaakcqWGqbaucqGGPaqkcqGH9aqpdaWcaaqaaiabigdaXiabicdaWiabicdaWiabd6gaUnaaBaaaleaacqWGWbaCaeqaaaGcbaGaemOta4eaaiabcYcaSiaaxMaacaWLjaWaaeWaaeaacqaIYaGmcqWGIbGyaiaawIcacaGLPaaaaaa@3EE7@

where *N *is the number of base pairs of the genome. Also,

*p*(~*P*) = 1 - *p*(*P*).

The *E. coli *K12 genome analyzed here has length *N *= 4639221 bp and approximately 4400 annotated genes. Assuming each gene has at least one promoter, and that all promoters for a gene lie in one 100 bp segment, we estimate the probability *p*(P) that a randomly chosen 100 bp segment contains a promoter to be *p*(P) = 0.095, so *p*(~P) = 0.905.

Next we need to find threshold values *T *that give acceptable values for the probabilities *p*(P | *D *<*T*) and *p*(~P | *D *<*T*). One wants the probability *p*(P | *D *<*T*) to be as large as possible, while keeping *p*(~P | *D *<*T*) small. As shown in Eqns 1a and 1b, these probabilities depend on the estimated number of promoter-containing and non-promoter-containing segments, and on the probabilities *p*(*D *<*T *| P) and *p*(*D *<*T *| ~P). Values for the latter probabilities can be found for the case of the summation SIDD parameter from the information in Figure 4A. There one sees that a threshold sum of *T =*308.5 gives a substantial probability *p*(*D *<*T *| P) that a promoter satisfies the threshold and a very small probability *p*(*D *<*T *| ~P) that a non-promoter does. Using this value one finds from the figure that *p*(*D *< 308.5 | P) = 0.236. From the SIDD profile of the whole genome we find there are 1356 regions where the sum Σ of the SIDD energies is less than 308.5. Thus, from Eqn 2a one finds that *p*(*D *< 308.5) = 1356*100/4639221 = 0.029. If we assume as above that there are 4400 promoter-containing regions, one for each gene, then Eqn 2b gives *p*(P) = 0.095. Substituting into Eqn 1a, we calculate that a 100 bp DNA fragment whose sum Σ of SIDD energies is less than 308.5 has probability of being a promoter *p*(P | *D *< 308.5) = 0.236*0.095/0.029 = 0.773. Similarly, we find in this case that *p*(*D *< 308.5 | ~P) = 0.01, so the probability of such a region not being a promoter is *p*(~P | *D *< 308.5) = 0.01*0.905/0.029 = 0.312.

We also performed similar calculations on the *B. subtilis *genome, which has length *N *= 4214630 bp, and approximate 4226 genes. Assuming again that each gene has one promoter-containing region, we find for this genome that *p*(P) = 4226*100/4214630 = 0.1003. When we choose the threshold value *T *= 316.5, we calculate that *p*(D < 316.5) = 1575 *100/4214630 = 0.0374. *p*(*D *< 316.5 | P) = 0.304 and *p*(*D *< 316.5 | ~P) = 0.008. Using these values in Eqns 1a and 1b, we find that the probability of a region being a promoter, given that it satisfies the threshold condition, is *p*(P | *D *< 316.5) = 0.815; and the probability of it being a non-promoter is *p*(~P | *D *< 316.5) = 0.193.

It is interesting to notice that the thresholds for the SIDD summation parameter are almost identical for *E. coli *K12 and *B. subtilis*. This seems unlikely to be a coincidence since these organisms are in different phyla, and have quite distinct genomic compositions, gene sets and environmental niches.

Two points must be borne in mind when interpreting these results, which are intended only to be illustrative. First, the values computed for these probabilities depend on an estimate for the number of promoters in the given genome. Because prokaryotic genes are often arranged into co-transcribing operons, it may not be correct that every gene has its own promoter. However, some genes in *E. coli *are known to have multiple promoters. And second, not all promoters are strongly destabilized. Because the SIDD method presented here has a small false positive rate, it can reliably find those promoters that are destabilized, but will not find those that are not.

### SIDD and -10 motifs together predict promoter and non-promoter sequences with high accuracy

As was described above, several types of DNA structural parameters, as well as -10 motif scores, have been suggested to differentiate promoters from non-promoter sequences, at least to some degree. One strategy for improving promoter predictions could be to combine together different variables that may not be highly correlated. We have calculated the correlation coefficient for each pair of these attributes over the entire collection of three training sets. Table 2 shows the results of this analysis. SIDD is moderately positively correlated with deformability and low thermostability, and moderately negatively correlated with -10 motif scores. High curvature is seen not to correlate with any other parameters used in this study.

We developed a linear classification function for discriminating promoter from non-promoter sequences in *E. coli *K12 that combines together multiple parameters [25]. (A description of how this was done is presented in the Methods section.) When all these variables were combined together, the resulting classifier achieved an accuracy of more than 82% on our test sets. However, a linear discrimination analysis that only included the SIDD sum parameter Σ and the -10 motif scores performed almost as well, attaining an accuracy of about 80%. Similar results were obtained when linear classifiers were developed for *B. subtilis*. (Data not shown.) The small loss of predictive accuracy that results when the other parameters are omitted indicates that, of the parameters examined here, SIDD and -10 motif scores are the two most effective attributes for differentiating promoters from non-promoters.

Sequence-based methods (including the PSSM techniques applied to -10 motif scores that were used here) are known to suffer high false positive rates. The high accuracy achieved by the linear classification functions developed here largely resulted from a dramatic decrease in the false positive rate that occurred when SIDD properties were included. Similar result were found for a eukaryotic promoter prediction program McPromoter [26]. There a combination of sequence information with several physical properties achieved a 30% reduction of false positives, when compared with the sequence model alone.

### Annotating promoter regions in microbial genomes using SIDD, either alone or combined with sequence motifs

**Figure 5:**
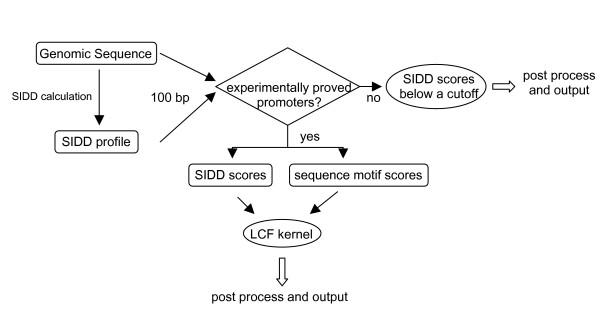
Schematic representation of a promoter region annotation strategy for microbial genomes.

The procedure schematically described in Figure 5 has been developed to annotate promoter regions in sample microbial genomes. Complete genomic sequences of more than 240 prokaryotic and archaeal organisms have been downloaded from the NCBI web site, and their SIDD profiles have been calculated. These SIDD profiles can be accessed through our database at [13]. To annotate the promoter regions in a given genome, our program calculates the sum of the SIDD energies and the minimum energy over a 100 bp window. This window is moved along the genome with an offset of 1 bp, and parameter values are calculated for each position. For those genomes that have enough experimentally characterized promoter sequences, we will additionally build PSSMs for the conserved motifs embedded in these sequences. The sum of the motif scores of the promoter sequences and non-promoter sequences are then calculated using these PSSMs. These scores, together with their correspondent SIDD scores (Σ and/or *G*_*m*_), are used as training sets for the linear classification function kernel to predict new promoter regions in the whole genome. Post-processing includes combining overlapping 100 bp potential promoter-containing regions into a single site prediction, and identifying potential transcriptional start sites. For those genomes that have insufficient experimental data to generate a meaningful PSSM, we will use SIDD as a sole predictor for discriminating promoters. Although the promoters predicted in this way may represent only a fraction of all the possible promoter regions in the genome, the present results suggest that they have high probabilities of being true.

We tested these approaches using the *E. coli *K12 genome. When SIDD scores were combined with -10 motif scores, 844 out of the 927 document TSSs were found to occur in the predicted 5272 potential promoter regions. When SIDD was used as a sole predictor and a threshold for Σ of *T *= 250 was used (this is a higher level of stringency than that used in the above Bayesian estimation), 340 documented TSSs were found in the predicted 1145 promoter regions. While the number of potential promoter regions was much smaller when SIDD was the sole predictor, the fraction of the predicted sites with experimentally documented TSSs doubled. Using either predictor, several instances were observed where multiple TSSs were located in a single predicted region. We expect the identification of these potential regulatory regions could enable further bioinformatics and experimental studies.

## Conclusion

In this report we show that the propensity to undergo stress-induced duplex destabilization (SIDD) is a distinctive structural attribute of prokaryotic promoter sequences. SIDD is not directly related to primary sequence alone, nor equivalent to the thermostability of the DNA double helix. Comparisons with other important DNA structural properties, and with conserved -10 motifs, show that SIDD is the best discriminator between promoter and non-promoter sequences in test sets derived from the *E. coli *K12 genome.

We have developed methods to identify promoter sequences in prokaryotic genomes that use SIDD either as a sole predictor or in combination with other DNA structural and sequence properties. The inclusion of SIDD properties is shown to greatly reduce the false positive prediction rates of predictions. So, for any given false positive rate, the true positive rate is higher when SIDD is included than when it is not. When applied to *E. coli *K12 and to *Bacillus subtilis*, the two experimentally best annotated bacterial genomes, our methods achieved comparably high levels of accuracy for both, and outperformed other published promoter prediction methods.

Because these organisms are from different phyla and their genomes have different nucleotide compositions, we suggest that this approach might be useful for other prokaryotes, for which experimental information may not be available. In all the microbial genomes we have investigated to date, strong SIDD sites have been found to be preferentially located in those intergenic regions that are known or inferred to contain promoters. The SIDD-based methods presented here have been implemented in a preliminary strategy for annotating promoter regions in complete microbial genomes. Although these methods cannot predict all the promoter-containing regions in a genome, they do find large sets of potential regions that have high probabilities of being true positives. These lists of predicted promoter-containing sites can be used as targets for experimental verification or further bioinformatic investigation. This approach could be especially valuable for those genomes about which there is limited experimental data.

## Methods

### Sequences

The *E. coli *K12 genome analyzed in this study is version M54, containing 4639221 base pairs. The locations of 927 experimentally characterized transcriptional start sites (TSS) were obtained from the Regulon Database [14]. The *Bacillus subtilis *genome analyzed here is the version submitted on 29 July, 2004, and containing 4214630 base pairs. Positions of 480 experimentally characterized TSSs in this organism were obtained from DBTBS [27]. In our analysis, the promoter regions were represented by the 100 base pair DNA fragments between positions -80 bp and +20 bp relative to a TSS. (This is 100 base pairs because in this scheme no base pair is given the number 0.) The promoter training set for *E. coli *K12 consisted of promoter regions of this size taken from 500 randomly chosen documented TSSs. The rest of the 427 promoter regions were used as a test set. The promoter training set for *B. subtilis *consisted of promoter regions from 250 randomly chosen documented TSSs. The rest of the 230 known TSSs were used to construct a promoter test set. The set of coding regions were selected as 100 base pair DNA fragments starting from +300 bp relative to (i.e. downstream from) the TSSs. The CON regions were chosen as 100 base pair DNA fragments centered in the middle of intergenic regions separating convergently transcribing ORFs. The coding and CON data sets each consisted of 500 randomly chosen regions of these types.

### Curvature calculations

The predicted values of DNA curvature were calculated for complete genomic sequences using the CURVATURE program [28], which creates a curvature map of the entire genome. For each base pair the curvature value (in curvature units, cu) corresponds to the curvature of the calculated path of a 121 bp segment centered at that base pair.

### Protein-induced deformability calculations

Values of the local protein-induced deformability were calculated complete genomic sequences using the dinucleotide model developed by Olson et al [29]. For each base pair in the genome, the deformability value is calculated as the average of the conformational volumes covered by its two neighboring DNA dimers in protein-DNA complexes.

### Thermostability calculations

The thermodynamic stability profiles of complete genomic sequences were calculated using the nearest-neighbor (NN) thermodynamics presented by SantaLucia et al. [30]. For each base pair in the genome, the value for its thermostability is calculated as the average of the opening energies for the two dinucleotides that contain it.

### SIDD profile calculations

The predicted values of the destabilization energy *G*(*x*) were calculated for complete genomic sequences using the method of Benham and Bi [11]. A superhelix density of *σ *= -0.06 was assumed. The destabilization free energy *G*(*x*) associated to the base pair at position *x *is the difference between the ensemble average free energy and the average free energy of those states in which base pair *x *is open. So it approximately corresponds to the incremental free energy needed to guarantee that this base pair is always open under the assumed superhelicity.

### DNA sequence conservation and position specific score matrix (PSSM) calculation

The conservation of multiple aligned sequences can be evaluated using the Shannon entropy of information theory [15]. Sequence motifs or conserved sequences are here evaluated using position-specific scoring matrices (PSSMs) calculated using the method of Durbin [3]. To calculate the sum and the number of high-scoring motifs in the training sequences, we used a threshold equal to the mean (-0.695) plus one standard deviation (1.214) of motif scores sampled in the randomly shuffled genomic sequence. About 55.2% of the documented -10 motifs in the promoter training set have their scores higher than this threshold, as compared with about 15.5% of the random sequences. Here, we consider the high-scoring motifs to be those whose scores are higher than the above threshold.

### Probability density estimation and statistical tests

The distributions of DNA structural properties or -10 motif scores in promoter regions, coding regions and CON regions were represented by their probability densities. The density for each property and type of region was evaluated using a normal kernel smoother at 100 equally spaced points covering the range of the data. The comparison of two distributions was made using the Kolmogorov-Smirnov two-sample test [22]. The null hypothesis for this test is that the two compared datasets are from the same continuous distribution.

### Linear discrimination analysis

We combined DNA structural properties (destabilization free energy, curvature, or thermostability) with -10 sequence motif scores into a linear discrimination model according to the method of Johnson and Wichern [25]. Because promoter identification is a two-class classification, it is implemented using Fisher linear discriminant analysis. This procedure finds a linear combination of the measures that provides maximum discrimination, in our case between promoter and non-promoters. It assumes that the training sets are normally distributed. To generate a score, we first let *w *= **S**^-1 ^*(*μ*_1 _- *μ*_0_). Here **S **is the pooled covariance matrix of the parameters, and *μ*_1 _and *μ*_0 _are the sample mean vectors of parameters for the positive and negative data sets, respectively. The vector *w *maximizes the ratio of inter-class variation of score to intra-class variation of score. The scores of a data point (vector) *x *associated to a member of a test set is calculated as the dot product D = x·w^T^. A data point is classified into class 1 if its score is D = c, or into class 0 if its score is D ≥ c; where c = w * (*μ*_1 _- *μ*_0_)^T^/2.

All the calculation relative to statistical tests and linear discrimination analysis were carried out using MATLAB 6.0.

The true positive rate, false positive rate, accuracy and precision are defined as follows (where *TP *= true positives, *FP *= false positives, *TN *= true negatives and *FN *= false negatives):

True positive rate = TPTP+FN
 MathType@MTEF@5@5@+=feaafiart1ev1aaatCvAUfKttLearuWrP9MDH5MBPbIqV92AaeXatLxBI9gBaebbnrfifHhDYfgasaacH8akY=wiFfYdH8Gipec8Eeeu0xXdbba9frFj0=OqFfea0dXdd9vqai=hGuQ8kuc9pgc9s8qqaq=dirpe0xb9q8qiLsFr0=vr0=vr0dc8meaabaqaciaacaGaaeqabaqabeGadaaakeaadaWcaaqaaiabdsfaujabdcfaqbqaaiabdsfaujabdcfaqjabgUcaRiabdAeagjabd6eaobaaaaa@348C@; False positive rate = FPTN+FP
 MathType@MTEF@5@5@+=feaafiart1ev1aaatCvAUfKttLearuWrP9MDH5MBPbIqV92AaeXatLxBI9gBaebbnrfifHhDYfgasaacH8akY=wiFfYdH8Gipec8Eeeu0xXdbba9frFj0=OqFfea0dXdd9vqai=hGuQ8kuc9pgc9s8qqaq=dirpe0xb9q8qiLsFr0=vr0=vr0dc8meaabaqaciaacaGaaeqabaqabeGadaaakeaadaWcaaqaaiabdAeagjabdcfaqbqaaiabdsfaujabd6eaojabgUcaRiabdAeagjabdcfaqbaaaaa@3470@; Accuracy = TP+TNTP+FN+TN+FP
 MathType@MTEF@5@5@+=feaafiart1ev1aaatCvAUfKttLearuWrP9MDH5MBPbIqV92AaeXatLxBI9gBaebbnrfifHhDYfgasaacH8akY=wiFfYdH8Gipec8Eeeu0xXdbba9frFj0=OqFfea0dXdd9vqai=hGuQ8kuc9pgc9s8qqaq=dirpe0xb9q8qiLsFr0=vr0=vr0dc8meaabaqaciaacaGaaeqabaqabeGadaaakeaadaWcaaqaaiabdsfaujabdcfaqjabgUcaRiabdsfaujabd6eaobqaaiabdsfaujabdcfaqjabgUcaRiabdAeagjabd6eaojabgUcaRiabdsfaujabd6eaojabgUcaRiabdAeagjabdcfaqbaaaaa@3E1C@;

Precision = TPTP+FP
 MathType@MTEF@5@5@+=feaafiart1ev1aaatCvAUfKttLearuWrP9MDH5MBPbIqV92AaeXatLxBI9gBaebbnrfifHhDYfgasaacH8akY=wiFfYdH8Gipec8Eeeu0xXdbba9frFj0=OqFfea0dXdd9vqai=hGuQ8kuc9pgc9s8qqaq=dirpe0xb9q8qiLsFr0=vr0=vr0dc8meaabaqaciaacaGaaeqabaqabeGadaaakeaadaWcaaqaaiabdsfaujabdcfaqbqaaiabdsfaujabdcfaqjabgUcaRiabdAeagjabdcfaqbaaaaa@3490@. Sensitivity = true positive rate, and specificity = 1 – (false positive rate)

## Authors' contributions

HW conceived and designed most of the strategies used here, with assistance from CJB. HW performed all the calculations and data mining. The paper was jointly written

## Supplementary Material

Additional File 1 Analysis of Bacillus subtilis. Analysis of the SIDD properties in the promoter regions of B. sublilis' genome Click here for file

## Figures and Tables

**Table 1:** SIDD is the most distinct variable that differentiates promoter from non-promoter sequences

	**Promoter region**	
	**vs.**	
	**Coding region**	**CON region**
**SIDD**	1.0308*10^-76 a^/ 4.0961*10^-72 b^	1.0398*10^-46 a^/ 2.5736*10^-44 b^
**Curvature**	2.4277*10^-15 a^/ 5.3737*10^-14 *c*^	5.3170*10^-5 a^/ 1.5965*10^-5 *c*^
**Deformation**	7.116*10^-63 a^/ 1.0000^ b^	1.2783*10^-31 a^/ 0.8567 ^b ^
**Thermo-Stability**	5.5028*10^-42 a^/ 0.4981 ^b ^	1.0527*10^-14 a^/ 0.9997 ^b ^
**-10 motif**	1.1882*10^-74 d^	2.6299*10^-30 d^

Each value in the table is the probability that the two distributions are the same, as found using the Kolmogorov-Smirnov two sample test.

a, sum of the values of the variables in the sequences; b, minimum value of the variable in the sequences; c, maximum value of the variable in the sequences; d, sum of the -10 motif scores of the sequences

**Table 2:** Correlation coefficients between structural parameters, -10 motif scores in promoter sequences

	**Sum of G(x) (Σ)**	**Curvature**	**Deformability**	**Thermo-stability**	**- 10 motif scores**
**Sum of G(x) (Σ)**		-0.0847	0.5194	0.3009	-0.5652
**Curvature**	-0.0847		-0.1499	-0.0207	0.1873
**Deformability**	0.5194	-0.1499		0.5317	-0.7546
**Thermo-stability**	0.3009	-0.0207	0.5317		-0.4175
**-10 motif scores**	-0.5652	0.1873	-0.7546	-0.4175	
