# Supplementary material for: Promoter prediction and annotation of microbial genomes based on DNA sequence and structural responses to superhelical stress
Source: BMC Bioinformatics. 2006 May 5;7:248. doi: 10.1186/1471-2105-7-248 (PMC1468432; doi:10.1186/1471-2105-7-248)
Supplement: Additional File 1 — Analysis of Bacillus subtilis. Analysis of the SIDD properties in the promoter regions of B. sublilis' genome [file 1471-2105-7-248-S1.ppt]

## Slide 1
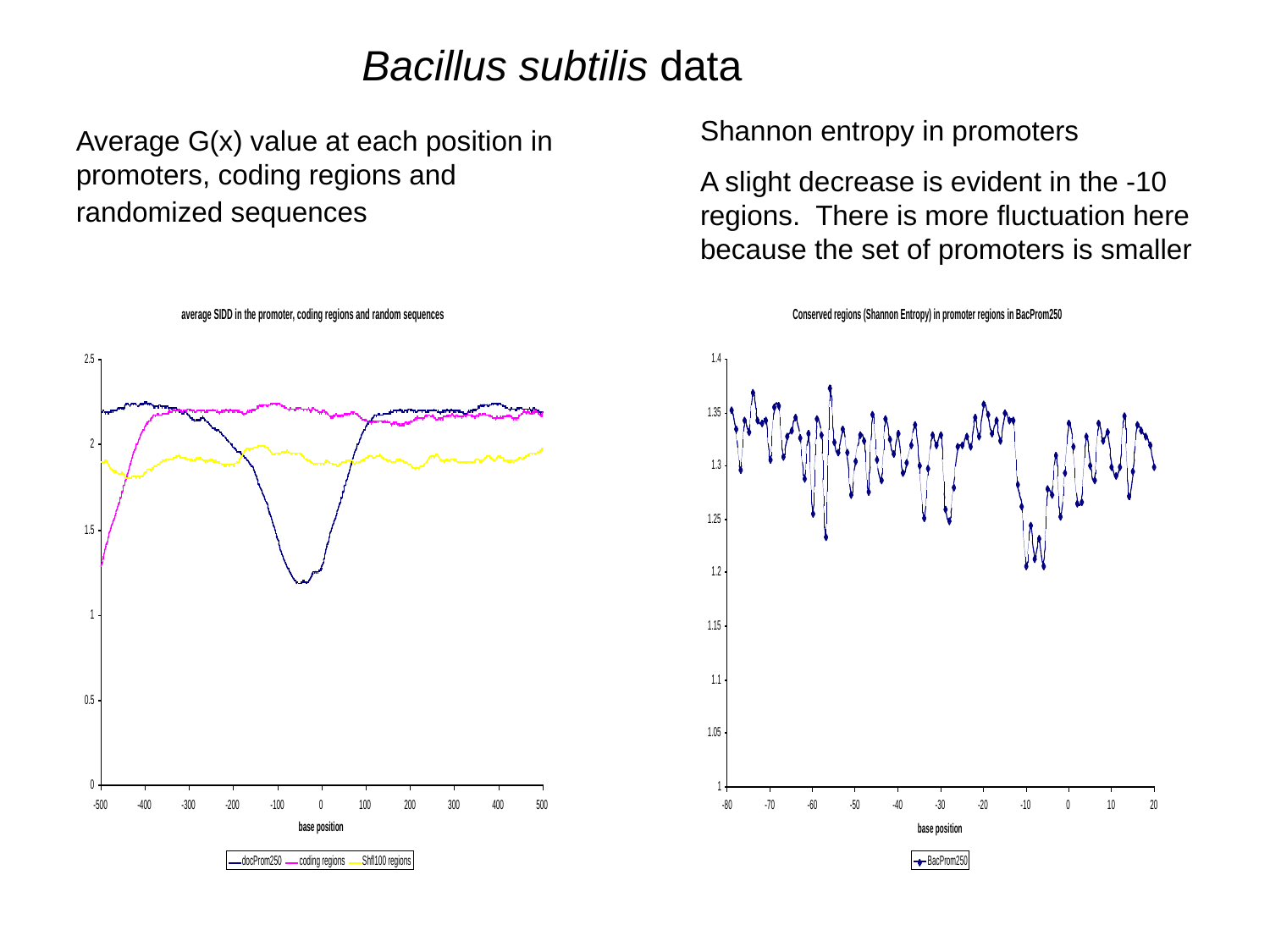

Bacillus subtilis data
Shannon entropy in promoters
A slight decrease is evident in the -10 regions. There is more fluctuation here because the set of promoters is smaller
Average G(x) value at each position in promoters, coding regions and randomized sequences

## Slide 2
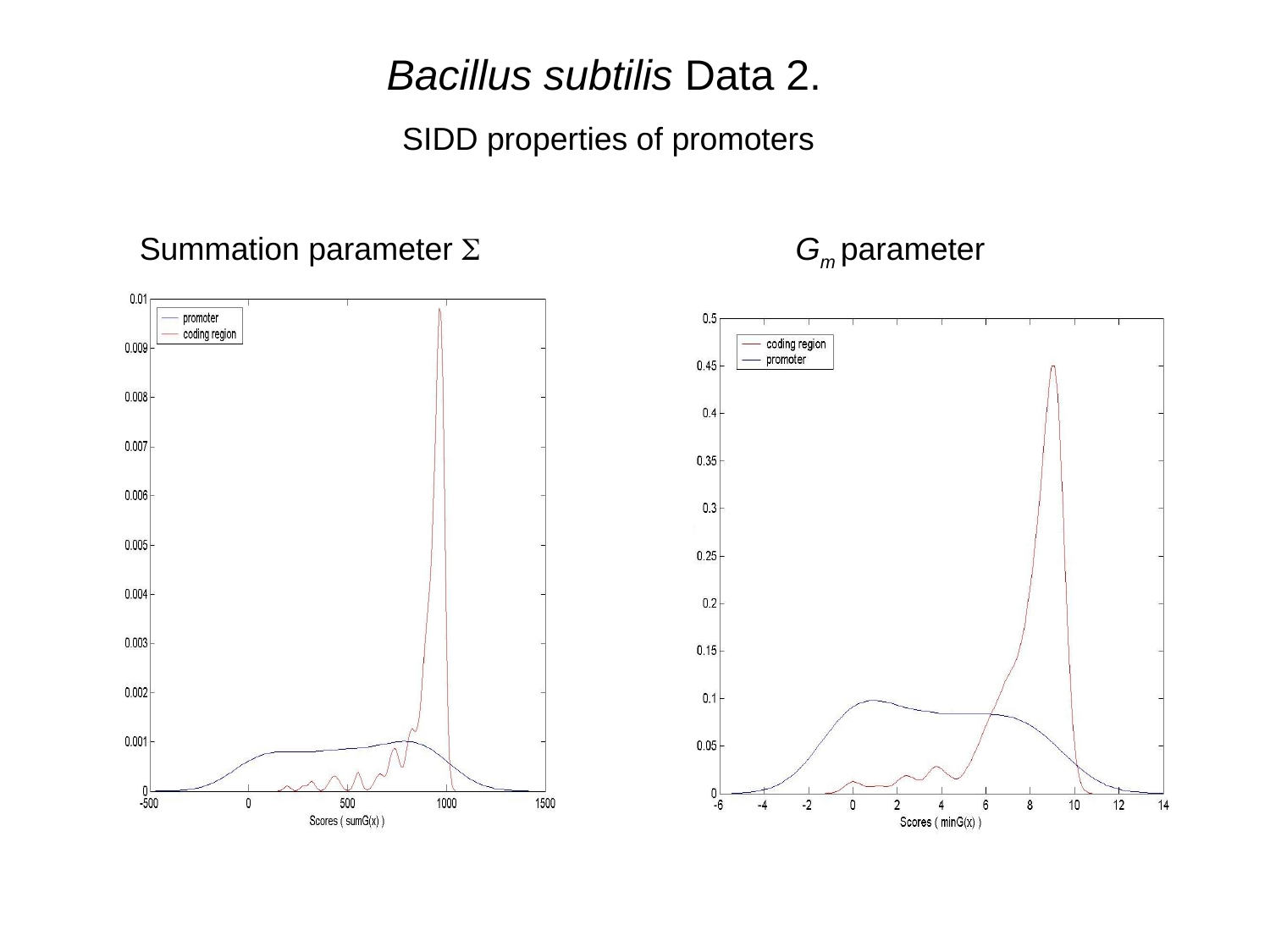

Bacillus subtilis Data 2.
SIDD properties of promoters
Summation parameter 
Gm parameter

## Slide 3
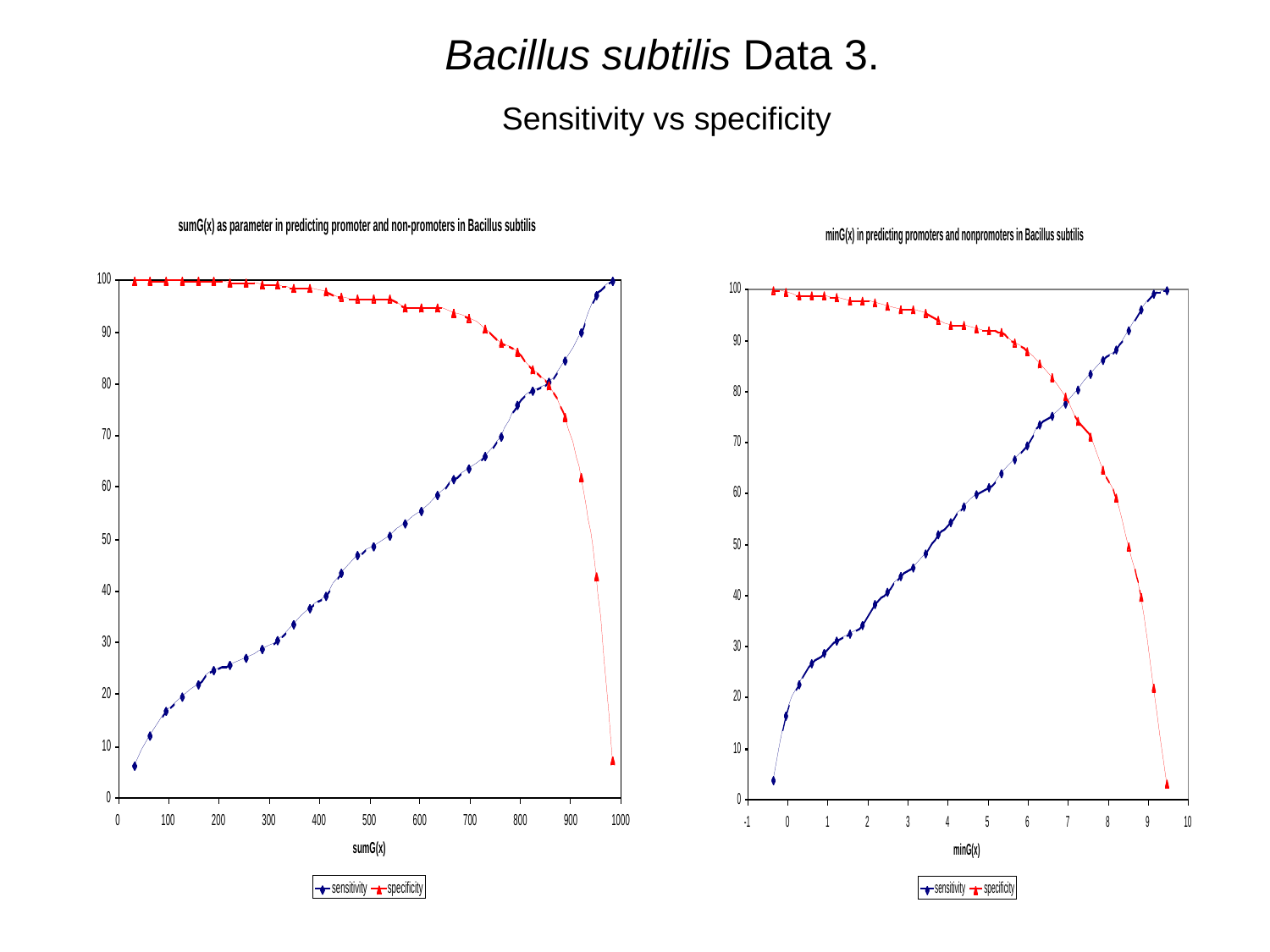

Bacillus subtilis Data 3.
Sensitivity vs specificity

## Slide 4
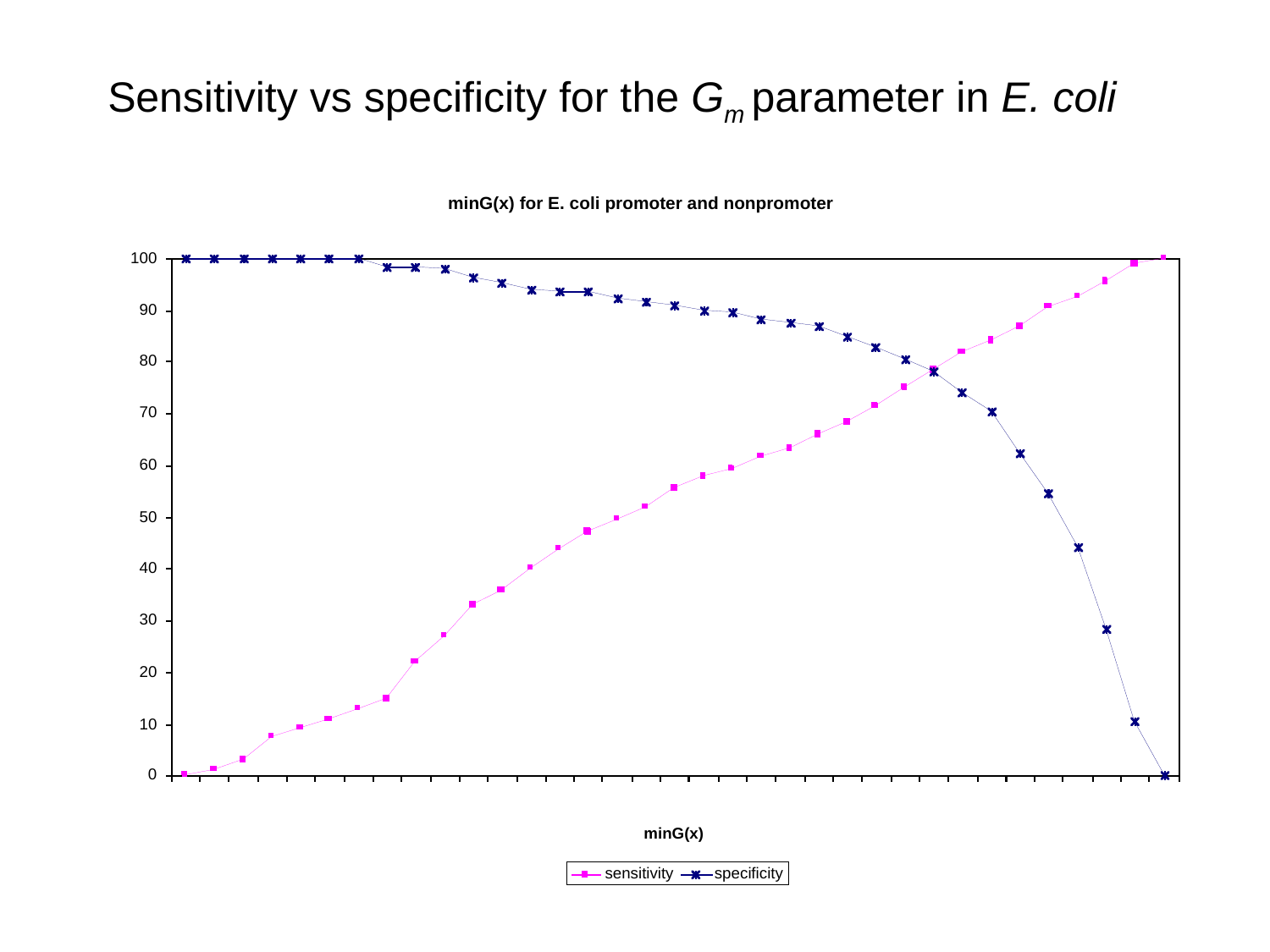

Sensitivity vs specificity for the Gm parameter in E. coli
